# Prevalence of TMJ Disorders among the Patients Attending the Dental Clinic of Ajman University of Science and Technology–Fujairah Campus, UAE

**DOI:** 10.1155/2018/9861623

**Published:** 2018-05-10

**Authors:** Kashef K. AlShaban, Zainab Gul Abdul Waheed

**Affiliations:** ^1^Faculty of Medical and Health Sciences, Emirates College of Technology, Abu Dhabi, UAE; ^2^Mint Dental Care LLC., Dubai, UAE

## Abstract

The objective of this study was to determine the prevalence of temporomandibular joint (TMJ) disorders (if any) among the patients attending the dental clinic (for routine dental treatment) of Ajman University of Science and Technology (AUST)–Fujairah campus, UAE, and its possible causes. A sample of 100 adult patients attending the dental clinic of AUST for different types of dental treatment were collected; the routine examination of the TMJ and possible disorders such as clicking, crepitation, limitation or deviation during mouth opening, or tenderness reveals that 41% of the sample experience varying degrees of disorders in the TMJ. Radiographs were taken if needed (panoramic radiograph). The information was collected and recorded for each patient through questionnaires.

## 1. Introduction

The proper function of (TMJ) depends upon the harmony of the different structures of the TMJ (including mandibular condyles, meniscus, glenoid fossa, ligaments, and muscles) that is well documented [1–4].

The TMJ continues in its function as usual until it is disturbed by external influences that affect the function of the joint, such as mechanical, psychological, occupational, and habits [5, 6].

The human body continues to try to repair its aggression; but if this continues, the body loses the ability to repair its aggression and the signs & symptoms begin to appear.

The signs and symptoms of TMD involve orofacial and preauricular pain, as well as limitation of mouth opening, TMJ bruit during function and displacement of articular disc [7].

Disorders of the (TMJ), and how people respond to them, vary widely and fall into three main categories:

1. Myofascial pain dysfunction syndrome involves tenderness or pain in the muscles that control jaw function.
2. Internal derangement of the TMJ involves anterior displacement of the meniscus with or without auto reduction, dislocated jaw, or injury to the condyle.
3. Arthritis refers to a group of degenerative/inflammatory joint disorders [8].

## 2. Materials and Methods

A random sample of 100 patients that attended the dental clinic for dental treatment underwent the routine examination of the TMJ, such as clicking, crepitation, limitation or deviation during mouth opening, and pain. 41% of these patients had varying degrees of TMD and were not aware of these disorders.

In the preliminary examination of the TMJ, we relied on the principles based on International RDC/TMD and the amendments thereto (version: 20 Jan 2014) [9].

This was done by individually examining each patient, taking a thorough history, and filling out a questionnaire for each patient, respectively.

Physical examination of the collected sample was done in two ways:

1. Lateral position: assessment of mandibular condylar movement by direct palpation over the joint while the patient opens and closes the mandible, at the preauricular area;
2. Posterior position: assessment of mandibular condylar movement by direct palpation of the mandibular condyle while the patient opens and closes the mandible, through the external auditory meatus.

A mandibular opening less than 35 mm (the distance between the edges of upper and lower incisors) is considered in our study as restriction of mouth opening.

A thorough history was collected from the samples on any history of previous trauma to the head and neck region, any previous long dental appointments that could be the reason for any of the TMJ disorders, or any habits or any occupation-related habits that could precipitate a TMD.

Along with the clinical examination of the patient, the aid of radiographs (if necessary) was also taken.

## 3. Results

**Figure 1:**
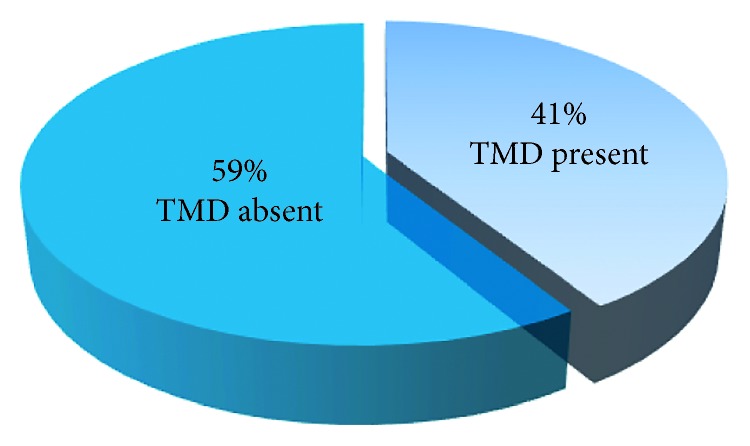
Pie chart demonstrating the presence and absence of TMD among the collected sample.

**Figure 2:**
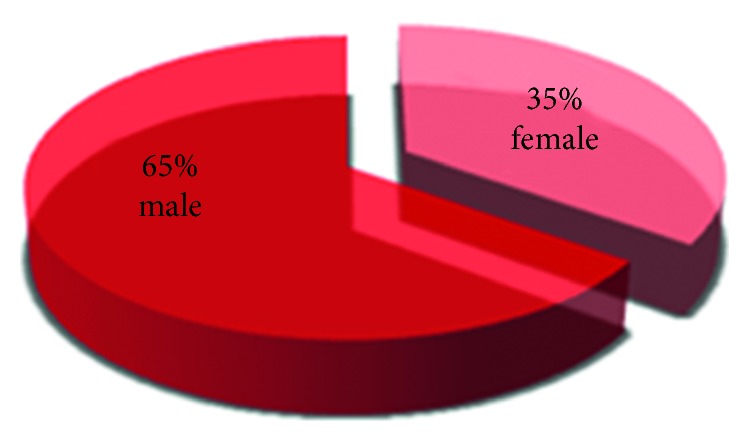
Pie chart demonstrating the percentages of male and female patients among the collected sample.

Among the randomly collected sample of a 100 patients, 41% (Figure 1) of the patient showed to have TMJ disorders out of which 65% were male patients and 35% were female patients (Figure 2).

**Figure 3:**
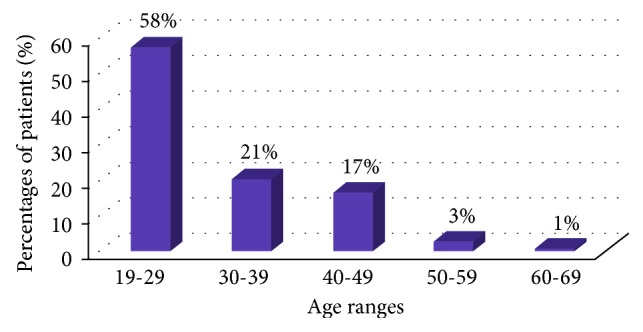
Bar graph showing the percentages of the collected sample of 100 patients and their respective age ranges.

Maximum percentage of patients (58%) was between the age ranges of 19–29 and the minimum (1%) were between the age ranges of 60–69 (Figure 3).

**Figure 4:**
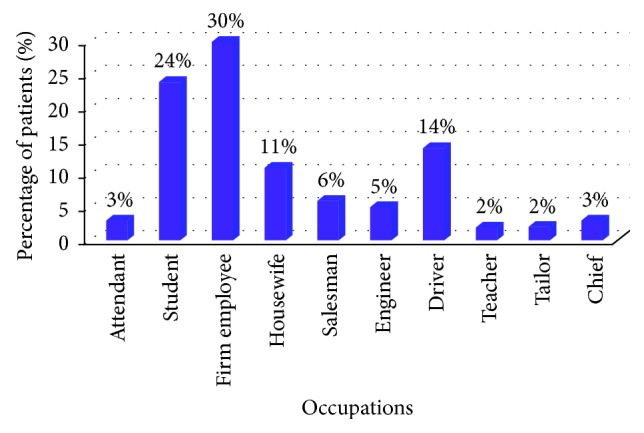
Bar graph representing the percentages of the collected sample of 100 patients with their respective occupations.

Patients were further categorized based on their occupation for evaluation of any occupation-related habits affecting the TMJ (Figure 4). The maximum numbers of patients were firm employees (30%), whereas the minimum numbers of patients were tailors and teachers (2%) among the total sample of 100 patients.

**Figure 5:**
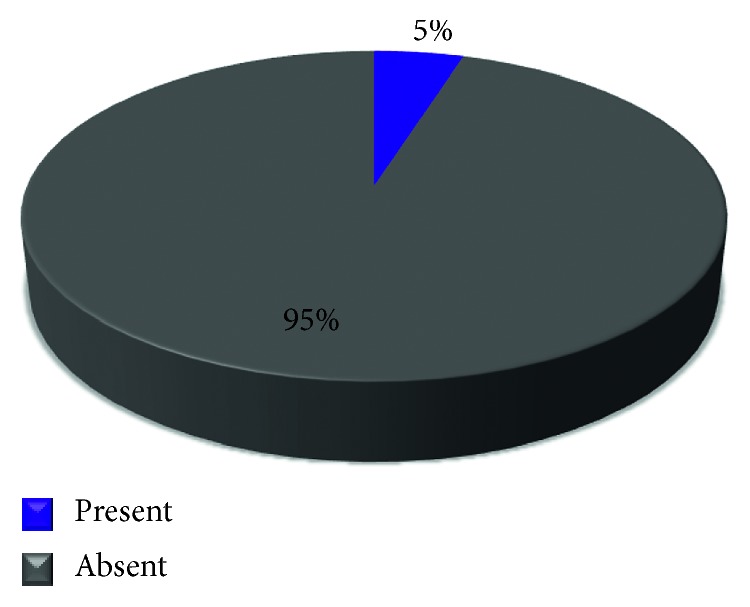
Pie chart representing the percentages of presence or absence of occupational habits in patients with TMJ disorders.

Among the 41% of the patients having the TMJ disorder, only 5% (Figure 5) of the patients had an occupation-related habit that could be a precipitating factor of the TMJ disorders.

The 41% of subjects that had the TMJ disorder were examined of various TMJ pathologies such as clicking, crepitation, limitation, deviation, and tenderness.

**Figure 6:**
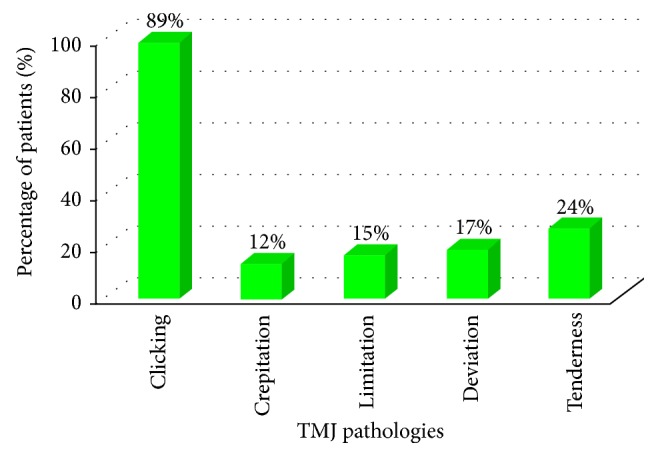
Bar graph showing the percentages of the patients having the different TMJ pathologies.

As a result of the examination, most percentage of subjects presented to have clicking (89%), and least percentage had crepitation (12%) (Figure 6).

**Figure 7:**
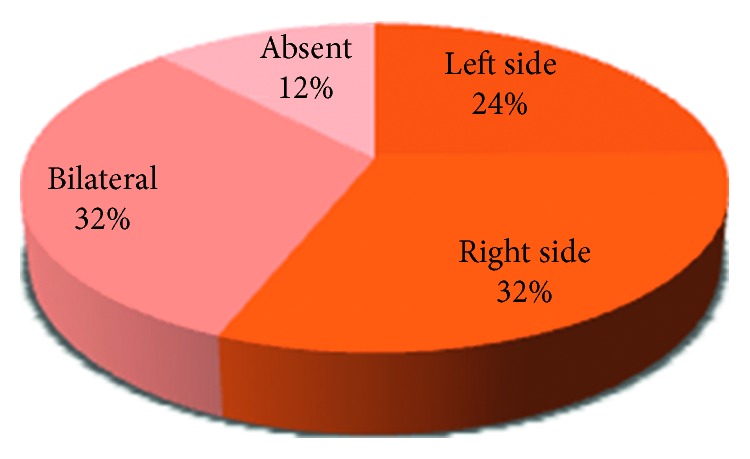
Pie chart demonstrating the percentages of patients with presence or absence of clicking on either side.

**Figure 8:**
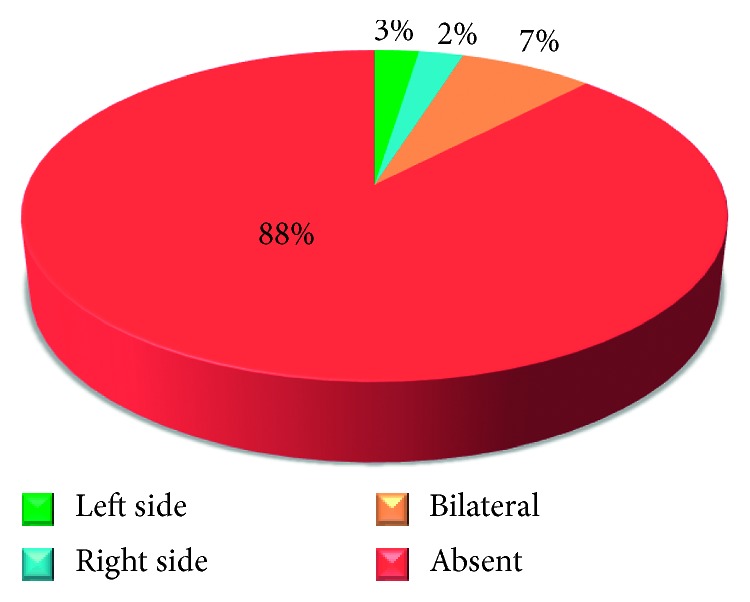
Pie chart demonstrating the percentages of patients with presence and absence of crepitation on either side.

**Figure 9:**
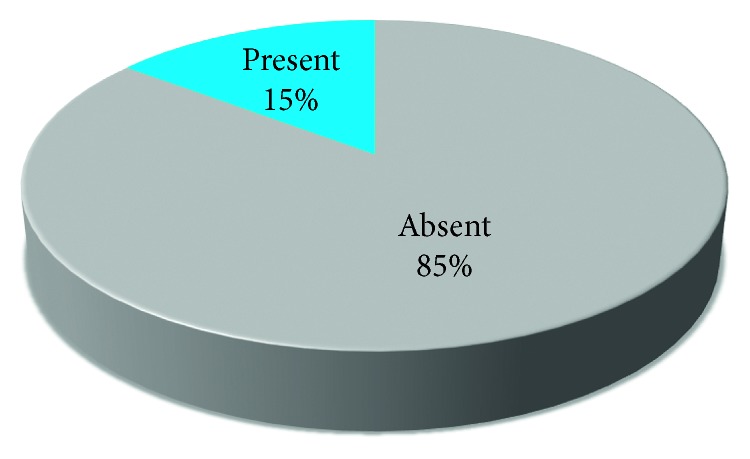
Pie chart representing the percentages of subjects with presence or absence of limitation during mouth opening.

**Figure 10:**
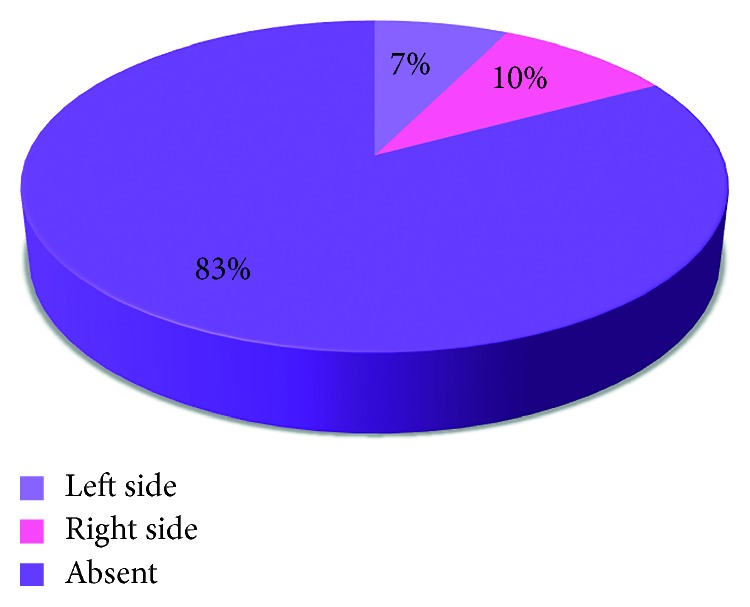
Pie chart demonstrating the percentages of patients representing presence or absence of deviation during mouth opening on either side.

**Figure 11:**
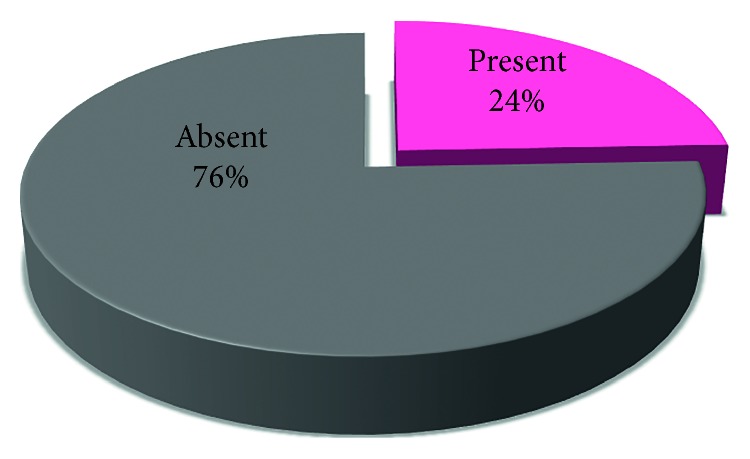
Pie chart representing the percentages of presence and absence of tenderness.

The involvement in TMJ Pathologies among the 41% of the patients either involved one side (unilateral) or both sides (bilateral) (Figures 7, 8, 9, 10, and 11).

**Figure 12:**
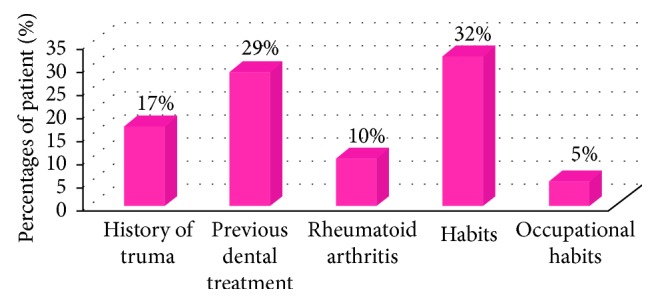
Bar graph representing the percentages of patients having different criteria.

The 41% of subjects were further inquired about five different criteria in order to achieve a conclusion of the possible causative factors of the TMJ disorders (Figure 12).

**Figure 13:**
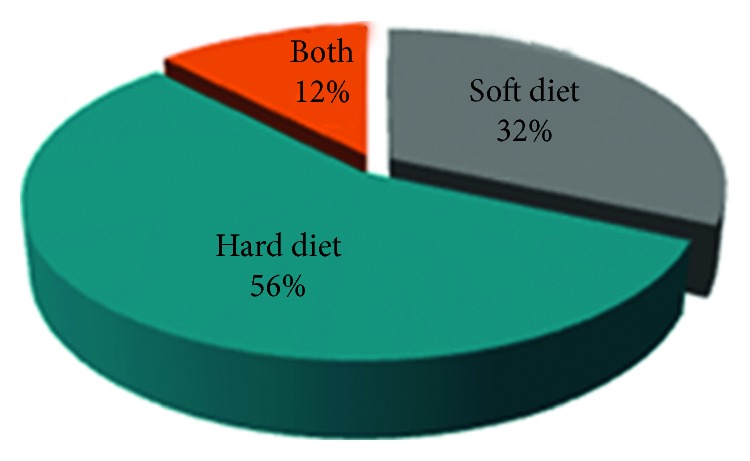
Pie charts demonstrating the percentages of patients whose diets are soft, hard, or both.

The subjects were inquired about the type of diet they consume and how it may affect the TMJ (Figure 13).

**Figure 14:**
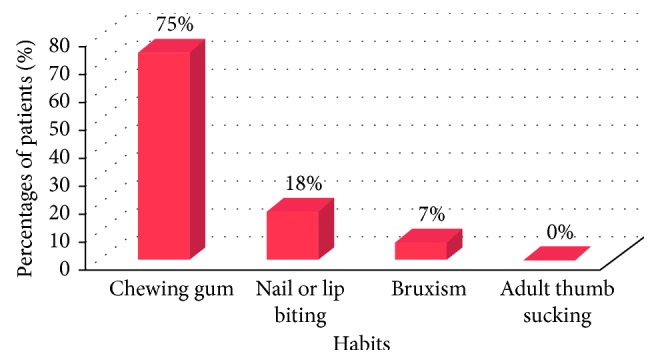
Bar graph representing the percentages of patients and their respective habits.

Figure 14 represents the percentages of patients and their respective habits.

## 4. Discussion

The research concluded that among the 41% of patients who had TMJ disorders, the most common TMJ pathology was clicking which was 89%, and most patients had either bilateral clicking (32%) or clicking on the right side (32%), followed by tenderness (24%), deviation (17%), limitation (15%), and crepitation (12%), as shown in related studies [6, 10, 11].

Further, the patients were questioned on different criteria with the goal of identifying the causative factors of the disorder:

- The gravest cause concluded from the research was habits (32%). Most patients had associated habits that resulted in TMJ disorders. Patients had habits like chewing gum (75%), nail or lip biting (18%), and bruxism (7%). Most patients were not aware of the severity of the habits on the TMJ, as shown in related studies [12–17].
- The second most common cause concluded was previous dental treatments (29%), specifying to a history of long dental appointments or previous orthodontic treatment [18].
- Patients were inquired about having any history of trauma to the head and neck area that may have had an impact on the TMJ. The results concluded that 17% of the patient had a history of trauma, as shown in related studies [19, 20].
- 10% of the patients had rheumatoid arthritis, as shown in related studies [21, 22].
- The lowest percentage was with occupational habits (5%). They were noticed in two patients, one was a teacher who had a history of clenching her jaws at the time of stress while teaching, and the other patient was a tailor having a history of biting off threads with his teeth.
- Patients were also inquired on the type of diet they consumed to access any possible relationship between the type of diet and TMJ disorder:

The results stated that most patients had a hard diet (56%) which could be a contributing factor in causing the disorders, as shown in related studies [23, 24]. Long-term consumption of hard diet has a direct impact on the TMJ; hence, the patients should be educated and motivated to discontinue the habit.

- In our study, the percentage of male patients who show TMJ disorders (65%) was higher than that of female patients (35%):

Lee et al. [6] reported in their study, the predominance of the male with TMJ disorders; Another study [10] concluded that no significant relationship between females and males regarding TMJ disorders; Some studies [11, 12, 17, 25] have shown clearly the predominance of the female with TMJ disorders; Hirsch et al. [26] reported in their study, the predominance of the female with TMJ disorders during pubertal development, but the diagnosis remains unknown.

More research is needed to try to elucidate the relationship between female hormones and TMJ disorders (cause and effect).

(i) Finally, the psychological pressure (state-anxiety) and disorders play a role in TMJ disorders [5, 6, 18, 27] (it is not included in our study). Most probably, the psychological stress results in bruxism (bite force) that increases the load on the masticatory system which is manifested by TMJ dysfunction.

While conducting the research, it was observed that majority of the patients had lack of awareness of having a TMJ disorder and the adverse effects they could experience in the future. Therefore, patient education is an important factor in the treatment of the disorder, and grave importance should be given to this subject in order to reduce the incidence of the disorders.

Patients who had habits should be educated and enlightened about the severe effects they are on to the TMJ and the further possible severities to the TMJ if the habits are not discontinued.

### 4.1. Treatment

This analysis demonstrated that most of the patients from the collected sample needed a nonsurgical approach towards the treatment (95%) such as splint therapy [28], physiotherapy [29], and transcutaneous electrical nerve stimulation [30]. The patients should be reassured and well informed regarding the at-home practices to relieve the symptoms.

Only 5% of the patient with long-term chronic conditions may need a surgical approach.

## 5. Conclusions

The research concluded that the incidence of TMJ Disorders is not uncommon among the patients attending the dental clinic of AUST–Fujairah campus.

TMD is a complex symptom, caused by multiple factors that are poorly understood. It affects people between 20 and 40 years old.

About 20% to 40% of the adult population is affected to some degree.

The harmony and balance between the masticatory system and the function are crucial to keep the TMJ complex healthy. If anything that disrupts this equation, the body tries to correct it but to a certain limit; when the abnormal functions continue, at that time the signs and symptoms appear.

So, we can conclude (according to our research) that the main etiology of the TMD is the abnormal functions of TMJ due to incorrect habits and practices, as demonstrated by our study.

Hence, more importance should be given towards educating the patient on how to avoid behaviors that are abusive to the TMJ as patient education is also a key to successful TMJ rehabilitation.

## Data Availability

Data supporting this research article are available from the corresponding author or first author on reasonable request.
